# Rapidly Progressing Myelodysplastic Syndrome Initially Presenting as Acute Leukemia

**DOI:** 10.7759/cureus.1096

**Published:** 2017-03-15

**Authors:** Sara Parylo, Adarsh Vennepurredy, Terenig Terjanian

**Affiliations:** 1 Internal Medicine, Staten Island University Hospital; 2 Hematology and Oncology, Staten Island University Hospital

**Keywords:** mds, myelodysplastic syndrome, acute leukemia, acute promyelocytic leukemia, leukemia

## Abstract

Myelodysplastic syndrome (MDS) refers to a group of various stem cell disorders, characterized by dysplastic and ineffective production in one or more cell lines. In general, MDS tends to present slowly over months to years and is commonly detected with routine bloodwork by primary care physicians. Patients may be asymptomatic and depending on age, comorbidities and risk classification of MDS may not require aggressive therapy. However, MDS carries the risk of progressing to acute leukemia over time. We present a case of rapidly progressive MDS in a previously healthy middle-aged female, originally presenting and treated as acute leukemia.

## Introduction

Myelodysplastic syndrome (MDS) refers to a diverse group of stem cell disorders which are categorized by ineffective hematopoiesis in at least one clonal cell line, leading to leukopenia, anemia, and/or thrombocytopenia. Symptoms of MDS depend on the underlying ineffective hematopoiesis, such as fatigue in light of anemia or purpura in light of thrombocytopenia. In general, MDS tends to have an indolent course. Many patients have no symptoms at all and are diagnosed with routine bloodwork. MDS may be triggered by previous chemotherapy, especially with alkylating agents, radiation, or benzene exposure, but it is frequently related to advanced age. There are about 4,000 per 100,000 MDS cases, making it the third most common hematologic malignancy, following non-Hodgkin lymphoma and multiple myeloma [1]. About 30% of MDS cases progress to acute leukemia [2]. We present a case of a patient with rapidly progressive MDS presenting similarly to acute leukemia. Informed consent statement was obtained for this study.

## Case presentation

**Figure 1:**
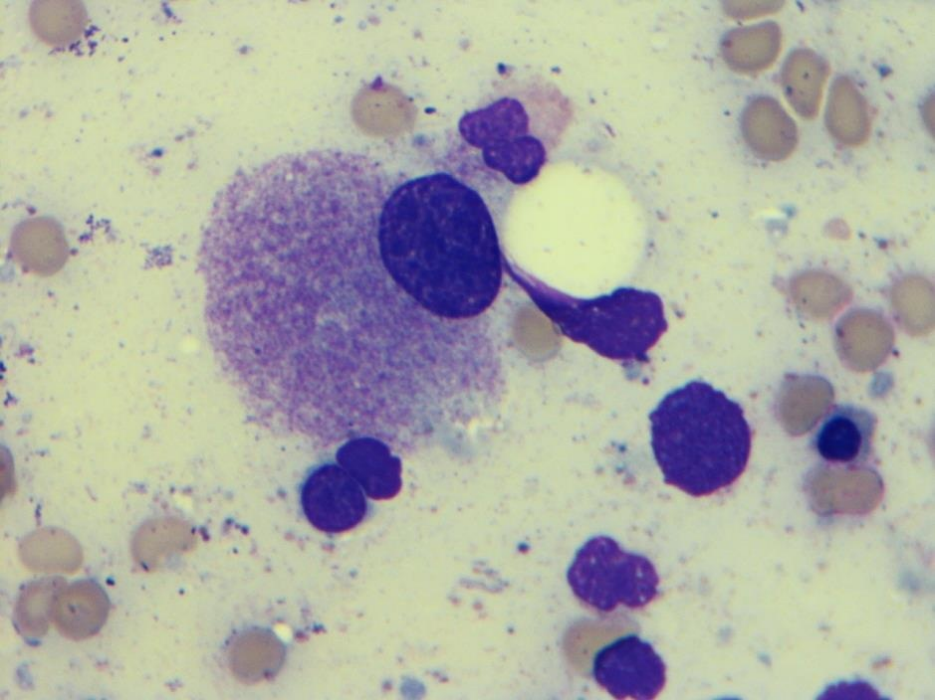
Monolobated megakaryocyte (1000X, Wright-Giemsa stain): Bone marrow aspiration smear shows monolobated megakaryocyte

**Figure 2:**
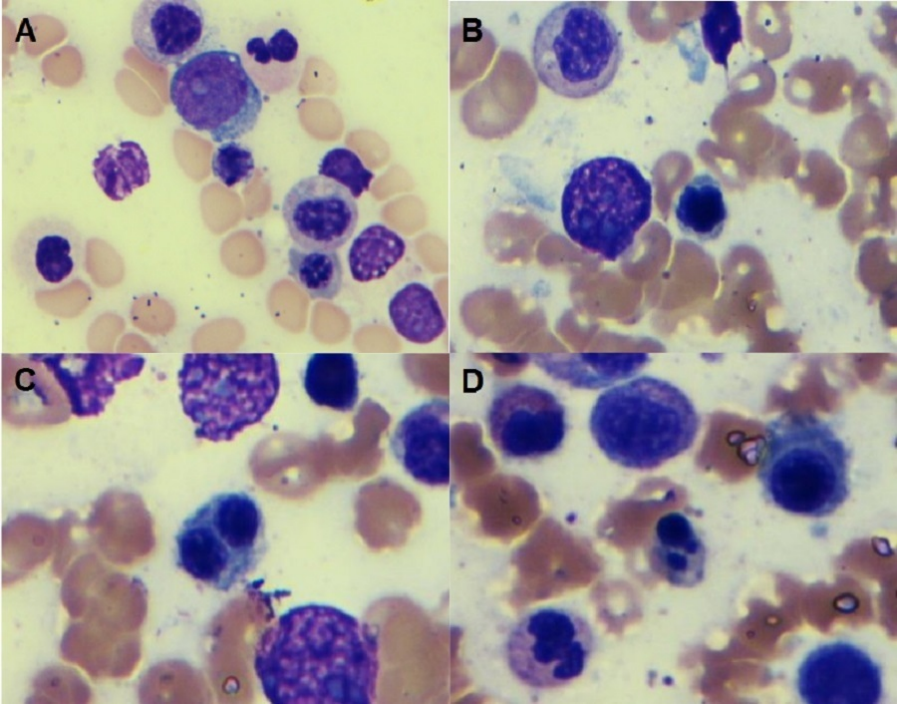
Granulocytic precursors (1000X, Wright-Giemsa stain): Bone marrow aspiration smear shows granulocytic precursors with slight hypogranulation (A) and dyserythropoiesis including nuclear contour irregularity (B), bi-nucleation (C), and Budding (D)

A 56-year-old female with no significant past medical history presented to the emergency room (ER) after being sent by her primary care doctor for decreased hemoglobin and hematocrit on bloodwork. The patient had been experiencing fatigue, dizziness, palpitations and episodes of near syncope for the previous four-six weeks. Five months prior to presentation, her complete blood count was completely within normal limits, with a WBC of 5.3 th/mm^3^, hemoglobin of 12.2 g/dL, hematocrit of 37.6%, and platelets of 197 th/mm^3^. ER bloodwork showed WBC of 4 th/mm^3^, hemoglobin of 6.9 g/dL, hematocrit of 21%, and platelets of 27 th/mm^3^. The patient was subsequently transfused with two units of packed red blood cells (PRBC). The results of the post-transfusion bloodwork were WBC of 5.6 th/mm^3^, hemoglobin of 8.6 g/dL, hematocrit of 26.1%, and platelets of 28 th/mm^3^. At that time, computed tomography (CT) of chest, abdomen, and pelvis was performed and did not reveal any clinically significant abnormalities. The patient was evaluated by a hematologist/oncologist, and bone marrow biopsy and flow cytometry were subsequently done (Figures 1-2).

The preliminary smear showed few atypical cells/blasts. Acute promyelocytic leukemia (APL) could not be excluded. Based on the risk of delaying APL treatment versus the risk of treatment toxicity, the patient was subsequently started on all-trans-retinoic-acid (ATRA), while complete results of flow cytometry and bone marrow biopsy were pending. ATRA was discontinued five days after being started, secondary to a normal flow cytometry. At this time, the patient was thought to have drug-induced bone marrow suppression secondary to receiving an antibiotic from the penicillin family for pneumonia-like symptoms several weeks earlier. The patient was subsequently discharged and instructed to follow with hematology/oncology in an outpatient setting. At the time of discharge, bloodwork showed WBC of 4.98 th/mm^3^, hemoglobin of 9.4 g/dL, hematocrit of 27.6%, and platelets of 29 th/mm^3^.

**Figure 3:**
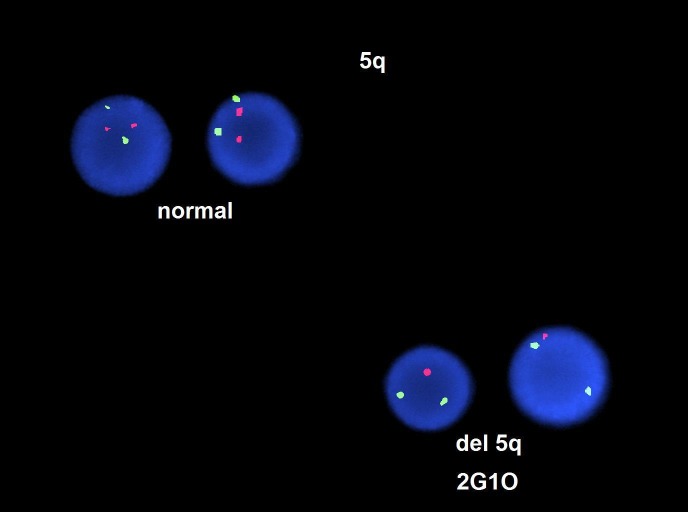
Fluorescence in-situ hybridization (FISH) showing chromosome 5q deletion

**Figure 4:**
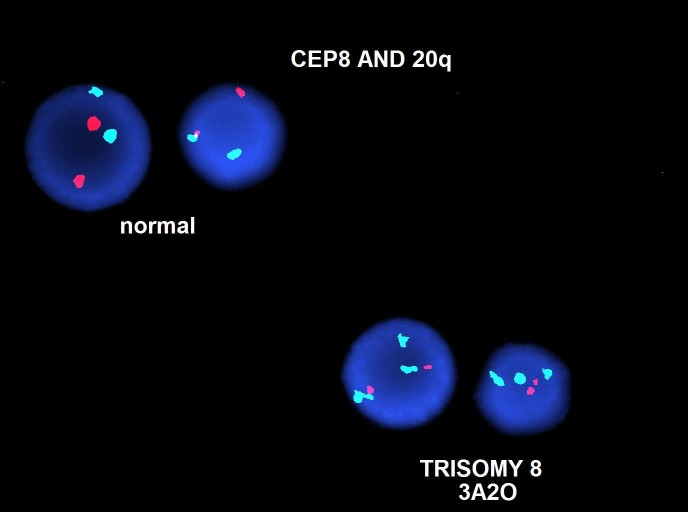
Fluorescence in-situ hybridization (FISH) showing trisomy eight

In the outpatient setting, she was subsequently diagnosed with high-risk MDS with chromosome 5q deletion and trisomy eight via fluorescence in-situ hybridization (FISH) (Figures 3-4).

She was then started on azacitidine treatment and supported with packed red blood cells (PRBC) and platelet transfusions as needed. The patient returned to the ER, 27 days after discharge with the complaints of fever, dry cough and fatigue. At the time of presentation, the patient had a WBC of 6.31 th/mm^3^, hemoglobin of 6.5 g/dL, hematocrit of 19.5%, and platelet count of 21 th/mm^3^, with an absolute neutrophil count (ANC) of 2.62 th/mm^3^. Chest x-ray showed new basilar opacities. CT scan of the chest was consistent with left lower lobe pneumonia, right hilar adenopathy and trace bilateral pleural effusions. The patient was started on cefepime and vancomycin for presumed healthcare associated pneumonia (subsequently switched to vancomycin, meropenem and levofloxacin when she continued to have fevers) and continued receiving azacitidine as per schedule. During the hospital course, the patient experienced a type IV hypersensitivity reaction to azacitidine, as well as to platelet transfusion, which were treated conservatively with diphenhydramine. At this time, the patient’s platelet counts did not respond appropriately to the transfusion. Direct Coombs test was performed and was negative along with work-up for underlying hemolysis and disseminated intravascular coagulation. The patient subsequently received an additional unit of PRBC and platelets and was discharged two days later with levofloxacin and cefpodoxime prescriptions and plans to continue azacitidine as a bridge to a bone marrow transplant.

However, the patient subsequently returned to the ER four days later with fevers (maximum temperature of 103 degrees Fahrenheit at home), dry cough and erythematous, pruritic papular rash (non-blanching on bilateral lower extremities and blanching on the back). Stevens-Johnson syndrome was ruled out. The patient was diagnosed with two separate rashes. The rash on the back was thought to be secondary to a drug eruption and the rash on the extremities was thought to be purpuric secondary to thrombocytopenia. The patient was treated with systemic steroids, along with topical steroids as needed for relief of pruritus. Antibiotics were stopped, since blood cultures, urine culture, and chest x-ray were negative for any evidence of infection. The patient received two more platelet transfusions without incident and was then discharged on famotidine, diphenhydramine, topical hydrocortisone, and prednisone. The patient continued to await bone marrow transplant for high-risk MDS.

## Discussion

MDS may be diagnosed with the symptomatic presentation of the specific underlying cytopenia but commonly is indolent initially and diagnosed incidentally with abnormalities on routine bloodwork in the primary care physician’s office [3]. This case illustrates rapidly progressive MDS within a period of two-three months presenting with symptomatic anemia and thrombocytopenia, along with a preliminary blood smear that could not exclude APL, leading to the decision to start the patient on ATRA therapy. The patient subsequently experienced numerous complications, including the need for numerous platelet and PRBC transfusions, fevers in light of broad spectrum antibiotic therapy, reactions to azacitidine and platelet transfusions and purpuric rash. Although unusual presentations of MDS have been reported, such as pulmonary leukocytoclastic vasculitis [4], diabetes insipidus [5], tumor lysis syndrome [6], bilateral sensorineural hearing loss [7], and bilateral acute angle closure glaucoma [8], it is extremely unusual to present as a rapidly progressive acute leukemia, leading to the fear of delaying an APL diagnosis and the rapid initiation of treatment with ATRA. Clinicians should be aware that MDS may present in this acute manner in which, case therapy should be promptly initiated and de-escalated as is clinically indicated.

## Conclusions

Although MDS has a variety of unusual presentations reported as case reports in the medical literature, it is extremely unusual to present as a rapidly progressive acute leukemia with symptomatic anemia and thrombocytopenia. The diagnosis of MDS was only made after a thorough analysis with peripheral smear, bone marrow biopsy, and flow cytometry and after five days of prophylactic ATRA therapy had been initiated. Clinicians should, therefore, have rapidly progressive MDS as a differential diagnosis when treating new acute leukemia.
