# Correction: Rapidly Progressing Myelodysplastic Syndrome Initially Presenting as Acute Leukemia

**DOI:** 10.7759/cureus.c8

**Published:** 2017-04-27

**Authors:** Sara Parylo, Adarsh Vennepureddy, Terenig Terjanian

**Affiliations:** 1 Internal Medicine, Staten Island University Hospital; 2 Hematology and Oncology, Staten Island University Hospital

When this article was initially published, the second author's name was misspelled as **Adarsh Vennepurredy**, however, the correct spelling is **Adarsh Vennepureddy**.

